# Dengue Infection Spectrum in Guangzhou: A Cross-Sectional Seroepidemiology Study among Community Residents between 2013 and 2015

**DOI:** 10.3390/ijerph15061227

**Published:** 2018-06-11

**Authors:** Jundi Liu, Yu Deng, Qinlong Jing, Xiashi Chen, Zhicheng Du, Tianzhu Liang, Zhicong Yang, Dingmei Zhang, Yuantao Hao

**Affiliations:** 1 School of Public Health, Sun Yat-sen University, Guangzhou 510080, China; liujd5@mail2.sysu.edu.cn (J.L.); dengyu9@mail.sysu.edu.cn (Y.D.); xiashi1991@sina.com (X.C.); duzhch3@mail2.sysu.edu.cn (Z.D.); tianzhu@bu.edu (T.L.); haoyt@mail.sysu.edu.cn (Y.H.); 2 Department of Infectious Disease, Guangzhou Centre for Disease Control and Prevention, Guangzhou 510440, China; jingqinlong@126.com

**Keywords:** dengue virus antibody, asymptomatic-infection, infection spectrum, seroepidemiology

## Abstract

The majority of dengue virus infections are asymptomatic, which could potentially facilitate the transmission of dengue fever and increase the percentage of sever dengue fever manifestations. This cross-sectional study explored the sero-prevalence of dengue virus infection in Guangzhou to clarify the infection spectrum. In total, 2085 serum samples were collected from residents of 34 communities. All samples were selected from a 200,000-sample database holding serum collected from community residents living in Liwan and Yuexiu districts of Guangzhou between September 2013 and August 2015, and 17 to 28 individuals of each age group were chosen per month. Dengue immunoglobulin G (IgG) and immunoglobulin M (IgM) antibodies were tested by enzyme-linked immunosorbent assay. Symptomatic infected individuals were identified via follow-up questionnaires. Among 2085 serum samples, anti-dengue IgG and IgM positive rates were 11.80% and 3.98%, respectively. The IgG antibody positive rate increased with age and was higher in poorly educated people than in highly educated people and in married individuals than in single individuals. Approximately 96.71% of dengue virus infections and an estimated 13.68% of the whole population were asymptomatic. Such high asymptomatic-infection rates have an impact on the local spread of dengue fever. Stricter surveillance, such as a network of rapid diagnostic laboratories, screening of residents in the epidemic season, and other integrated control measures are necessary.

## 1. Introduction

It is estimated that 2.5 billion people worldwide are at risk of dengue fever infection, and at least 100 countries have suffered from dengue fever outbreaks [1,2]. Approximate 390 million dengue fever cases occur annually across the world, and about 250,000 cases involving significant or severe manifestations such as dengue hemorrhagic fever (DHF) and dengue shock syndrome (DSS) occur worldwide [1]. However, dengue virus infection usually occurs with no clinical or mild manifestations, referred to as asymptomatic infection [3]. Several studies reported that a high percentage of dengue fever infections were asymptomatic in both endemic and non-endemic areas [4,5]. Therefore, among the estimated 390 million dengue fever cases, only 96 million cases show clinical manifestations and the rest of the 294 million cases are asymptomatic infections [6,7]. Asymptomatic dengue infection is usually not detected by surveillance programs. Therefore, the asymptomatic population is easy to be neglected by the monitoring system for dengue fever. Furthermore, asymptomatic dengue infected population might continue to live and work without protective measures. Thus, high rates of asymptomatic infection in specific areas might have a great impact on local dengue virus transmission, as well as severe outcomes when secondary infections occur. 

From 1978 to 2013, a total of 14,831 confirmed cases of dengue fever were reported in Guangzhou city (23°08′ N, 113°16′ E) [8]. During 2011 to 2013, the number of confirmed cases increased significantly every year, and numbers of reported cases were highest for Liwan District, Yuexiu District, and Haizhu District, accounting for 40.63%, 26.25%, and 12.54%, respectively, of total cases [9]. Additionally, in 2014, there were 37,340 cases reported when Guangzhou was identified as having a dengue fever outbreak [10].

Apparently, most of the dengue cases live in Liwan District and Yuexiu District [11] and the majority of the population is native to Guangzhou. Therefore, the two old town areas are good representatives of Guangzhou for dengue study. Recently, several dengue fever studies on the relationship between meteorological variables and dengue prevalence and spatial and temporal characteristics of dengue outbreak in Guangzhou were published [12,13,14]. However, limited information about the dengue infection spectrum in Guangzhou was found.

In order to clarify the spectrum of dengue virus infection in Guangzhou and to provide supporting evidence for predicting epidemic trends and developing intervention measures, the sero-prevalence of dengue virus infection was estimated by immunoglobulin G (IgG) and immunoglobulin M (IgM) detection among the community population in Guangzhou in this study. The dengue IgG antibody will be produced slowly, with low titres 8–10 days after having a fever. Therefore, the dengue IgG antibody cannot be detected in the early period of infection; however, it is detectable in the late or recovery period and can be measured for years after an infection, serving as an evidence of dengue infection in the past. The dengue IgM antibody is detected easily about five days after a fever and lasts 2–3 months [15].

Therefore, any detection of IgM antibodies provides evidence of a recent dengue fever infection. Based on a 200,000-sample database holding serum collected from community residents living in Liwan and Yuexiu districts of Guangzhou between September 2013 and August 2015, 2085 serum samples were tested for dengue IgG and IgM from residents of 34 communities. According to the results of IgG and IgM tests, along with clinical diagnosis and reported records in the National reporting system for notifiable infectious diseases, the infection spectrum of dengue virus in Guangzhou was estimated.

## 2. Materials and Methods

### 2.1. Sample Collection

Serum sample demographic information was obtained from an approximate 200,000-sample database established by the National Science and Technique Major Project of China—the 12th Five-Year Plan: Research on Prevention and Control of Human Immunodeficiency Virus (HIV) and Hepatitis B Virus (HBV) in Guangdong Province project, which collected serum from community residents living in Liwan and Yuexiu districts of Guangzhou from September 2013 by recruiting volunteers through residents’ physical examination in community health service centers and schools. Among the 200,000 samples, the male to female ratio was 0.47:1. The proportions of age groups were 16.68%, 18.1%, 36.63% and 28.59% (under 19 years old, 19 to 40 years old, 41 to 65 years old, and above 65 years old, respectively). In addition, 7.6% of the participants were illiterate, and the percentages of the individuals with other education degrees (primary, junior high school, senior high school and diploma and over) were 32.27%, 18.89%, 20.04% and 6.71%, respectively. Moreover, married individuals composed the largest percentage (66.63%) of the population. Therefore, for better representativeness, 17 to 28 samples from the database of each age group (under 19 years old, 19 to 40 years old, 41 to 65 years old, and above 65 years old) were chosen per month between September 2013 and August 2015 to test for the dengue antibody. In total, 2085 serum samples were selected.

The present research was approved by the Institutional Review Board of the School of Public Health at Sun Yat-sen University (L2017030), in accordance with the guidelines for the protection of human subjects. Participants provided written informed consent after being briefed on the purpose of the study and of their right to keep information confidential. Written consent was obtained from all study participants or their guardians.

### 2.2. Enzyme Immunoassay Test

Dengue IgG and IgM antibodies to dengue viruses 1, 2, 3 and 4 were tested via enzyme-linked immunosorbent assay (ELISA) using Dengue IgG Indirect ELISA Test kits (LOT: 01P20A006, Inverness Medical/Panbio, Windsor, Australia) and Dengue IgM Capture ELISA Test kit (LOT:01P30A002, Inverness Medical/Panbio, Windsor, Australia) in the central laboratory of the School of Public Health, Sun Yat-sen University. As described in the instructions of the kits, a sensitivity of 97.9% and a specificity of 100% were estimated by using IgG indirect ELISA assay, compared to a sensitivity of 94.7% and specificity of 100% for IgM ELISA. High specificity and sensitivity ensured high credibility of these methods. The ELISA samples with inconclusive results were retested by using the colloidal gold test according to the Dengue Fever IgG and IgM RapiCard protocol (LOT: DEN141001, Inverness Medical/Cortez, Calabasas, CA, USA), and the sensitivity and the specificity of the kits were 91.43% and 98.85%, respectively. Absorbance of samples was tested by an absorbance reader (ELx800, BioTex, Winooski, VT, USA) at a wavelength of 450 nm, with a reference filtering of 600–650 nm.

### 2.3. Spectrum of Dengue Infection Identification

All of the individuals detected as IgG antibody positive, IgM antibody positive, or positive for both IgG and IgM antibodies were asked to recall if they were once diagnosed as dengue fever and provide the diagnosis hospital information. At the same time, the personal identification information of the positive cases was compared with all the reported dengue cases in the national reporting system for notifiable infectious diseases from the Guangzhou Center for Disease Control and Prevention to verify information provided by the subjects. Those with hospital diagnosis and/or reported as dengue cases for the same specified study period from 2000 to 2015 were identified as symptomatic-infection.

Asymptomatic-infected cases were defined as individuals who were at least one kind of antibody positive, but without dengue fever diagnosis or dengue reported cases for the same specified study period from 2000 to 2015. The spectrum of dengue infection in Guangzhou was established by calculating the rate of total current infected, asymptomatic-infected and symptomatic-infected cases. The rate of total current infected cases was estimated by using IgM sero-positives, IgG sero-positives or both sero-positives as the numerator and all samples as the denominator; the rate of asymptomatic-infected cases was estimated by using asymptomatic-infected cases as the numerator and all samples as the denominator; the rate of symptomatic-infected cases was estimated by using symptomatic-infected cases as the numerator and all samples as the denominator.

### 2.4. Data Analysis

Data analysis was conducted using SPSS20.0. Significant differences were analyzed via descriptive statistics and the Chi-Square test. The significance level was 5% (*α* = 0.05). Odds ratio and 95% confidence intervals (CI) were calculated for variables with significant differences.

## 3. Results

### 3.1. The Distribution of Residents Whose Serum Samples were Collected

**Figure 1:**
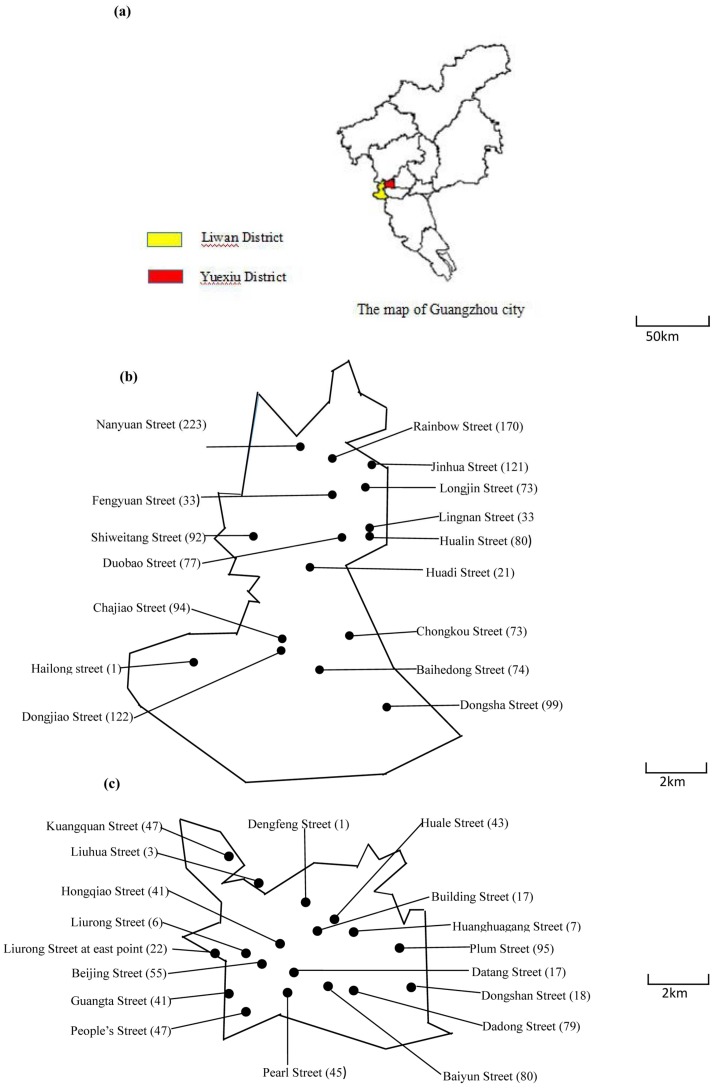
The distribution of residents whose serum samples were collected. The location of community health service centers collecting serum samples from administered residents. Numbers in parentheses indicate how many samples were collected. (**a**), The situation of Liwana District and Yuexiu District. (**b**), The distribution of sampling community health service centers in Liwan District. (**c**), The distribution of sampling community health service centers in Yuexiu District.

Figure 1 shows that 1386 serum samples were collected from 16 community health service centers in Liwan District, and 699 serum samples were collected from 18 community health service centers in Yuexiu District.

### 3.2. The Population Distribution of Immunoglobulin G and Immunoglobulin M

The demographic information of the 2085 serum samples shown in Table 1 illustrates that most participants were female (63.4%), married (60.9%) and between 41 and 65 years old (32.6%). The dengue IgG positive rate was 11.80% (95% CI: 10.41–13.18%). The dengue IgM positive rate was 3.98% (95% CI: 3.14–4.82%). Also, there were 34 serum samples positive for both dengue IgG and IgM, so the current infection rate of both positive samples was 1.63% (34/2085) (95% CI: 1.09–2.17%). The IgG antibody positive rates were significantly different among age groups, educational status and marital status (*p* < 0.05). The IgG antibody positive rates increased with age. Compared with those under 19 years old, people were 3.13 times, 4.45 times, and 25.45 times to be IgG positive in the 19–40 age group, 41–65 age group, and over 65 years old, respectively. Besides, compared with illiterate people, those with senior high school and diplomas or higher education had lower risk of being IgG positive (0.53 times and 0.52 times, respectively). In addition, the risk of dengue IgG positive of the married group, the widowed group, and the divorced group was 6.01 times, 15.73 times, and 7.43 times as high as that of the single group. However, there was no statistical significance of an IgG antibody positive rate between male and female (*p* = 0.212), as well as IgM antibody positive rate between gender, age group, marital status or educational status. Dengue IgG positive rates in Liwan District and Yuexiu District had no statistical difference (*p* = 0.939). The IgG antibody positive rates of those under 19 years old and who were illiterate were statistically different in Liwan District and Yuexiu District. For those under 19 years, the risk of the dengue IgG positive rate of the group in Yuexiu District was 5.55 times as high as that in Liwan District (OR = 5.55; 95% CI: 1.06–29.00; *p* < 0.05). Meanwhile, for illiterate people, the risk of being dengue IgG positive in Yuexiu District was 2.60 times as high as that in Liwan District (OR = 2.60; 95% CI: 1.02–6.63; *p* < 0.05). Dengue IgM positive rates in Liwan District (4.40%) were slightly higher than those in Yuexiu District (3.15%), but there was no statistical difference (*p* = 0.167). The females in Yuexiu District had lower risk of being IgM positive than their counterparts in Liwan District (OR = 0.49; 95% CI: 0.25–0.93; *p* < 0.05).

### 3.3. The Time Distribution of Immunoglobulin G and Immunoglobulin M

As shown in Table 2, the time periods were divided into four groups according to the outbreak period. Both the IgG and IgM antibody positive rates were different across the four periods in Guangzhou (*p* < 0.05). Further analyses indicated that the IgG antibody positive rate (17.00%) and IgM antibody positive rate (9.35%) in the September to December 2014 group were the highest among the four periods. Simultaneously, no significant difference was observed among the other three groups for both IgG and IgM antibody positive rates. From September to December 2014, when Guangzhou was considered as having a dengue fever outbreak, the respective risk of being dengue IgG positive and IgM positive was 1.73 times and 5.04 times higher compared with the same period in 2013. At the same time, the IgM positive rate increased from 2.01% to 9.35% between 2013 and 2014 but declined after December 2014.

### 3.4. Spectrum of Dengue Virus Infection in Guangzhou

The infection rate (dengue IgG, IgM antibodies or both positive) was 14.15% (295/2085; 95% CI: 12.65–15.65%). Among the 295 positive cases, 243 samples were followed up and eight individuals were diagnosed as having symptomatic dengue virus infection from 2000 to 2015 in Guangzhou hospitals. Therefore, among dengue infection cases, 3.29% (8/243; 95% CI: 1.03–5.55%) were symptomatic infection, and 96.71% (235/243; 95% CI: 94.44–98.97%) were asymptomatic. In the general community population, the symptomatic infection rate was estimated as 0.47% (295 × 3.29%/2085; 95% CI: 0.18–0.78%), and the asymptomatic infection rate was estimated as 13.68% (295 × 96.71%/2085; 95% CI: 12.19–15.14%). The ratio between symptomatic-infected and asymptomatic-infected cases in the community population was estimated as 1:29.

## 4. Discussion

The dengue IgG antibody rate was 11.80% in Guangzhou, which was lower than in the Dominican Republic, in which it was reported that 98% of blood donors and 56% of children were positive for dengue IgG [16,17], and also lower than in Singapore and Pakistan, where the dengue IgG positive rate was 59% and 67.2%, respectively [18,19]. Because dengue infection is not considered to be endemic in Guangzhou, the incidence of infection is relatively low compared with these tropical countries which were all epidemic foci that had serious prior local outbreaks [14,20]. However, there is an increased trend toward the incidence of dengue fever in recent years in Guangzhou [21]. As the third largest city in China, Guangzhou has frequent import and export activities and connections with Southeast Asian countries, which increases the chance of local people being exposed to dengue virus infection. Meanwhile, the prevalence of dengue IgG antibodies increased significantly with age, and people over 65 years old had the highest rate, which is most likely attributable to the increased possibility of exposure to dengue virus over their lifetimes, that is, the effect of accumulation of IgG antibodies over time. Another interesting phenomenon is that illiterate people had higher IgG antibody levels, which might be ascribed to their tendency toward working outside without proper prevention of awareness and measures, increasing their exposure to mosquitoes. Also, married individuals had a higher prevalence of dengue IgG antibodies than single individuals. The reason might be that married individuals share the same space with family members and are more likely to be infected by their infected families through the bites of mosquitoes. On the contrary, there was no statistical significance of an IgM antibody positive rate among age groups, marital status or educational status. This might be due to a small number of dengue IgM positive samples. After all, the small sample size could result in a higher type I statistical error rate, and it also could lead to a widening of the range of the odds ratio of the confidence interval.

The rate of both dengue IgG and IgM antibody positive cases was 1.63%. The serum samples positive for both antibodies might be secondary infected persons, and secondary infection is one of the risk factors for DHF/DSS [22]. Whether the 34 serum samples are primary infection or secondary infection is uncertain in this study, and further study is required.

The positive IgG antibody rates in Yuexiu District were statistically higher than in Liwan District in the age group under 19 years old and those who were illiterate. The cause of the result might be that there is a larger proportion of people under 19 years old and a low proportion of illiteracy in Yuexiu District, which is due to more primary and secondary schools in Yuexiu District than in Liwan District. Meanwhile, people under 19 years old are one of the susceptible populations to dengue virus infection as the study showed [23]. In addition, the positive IgM antibody rates in the two districts were statistically different in females, which might be due to the small simple size.

In this study, compared to the corresponding period of the previous year (September to December 2013), both dengue IgG antibody and dengue IgM antibody positive rates in Guangzhou significantly increased from September to December 2014, which is due to the dengue fever outbreak at that time. However, only the IgM antibody positive rate remained significantly higher from January to August 2015 compared to the same month period in 2014 in Guangzhou, even though the incidence did decrease after the epidemic situation was under control after December 2014. These findings suggested that seasonal dengue fever control in Guangzhou requires improvement, although emergency control management during and after the outbreak was well implemented.

As Thai et al. [24] and Egger’s [25] studies suggested, adults were more likely to develop clinical (symptomatic) dengue infection than children [26]. These eight diagnosed cases in Guangzhou were all adults, which is consistent with such a hypothesis. The majority of infections in Guangzhou were transmitted silently, particularly between children [27]. Therefore, many asymptomatic-infected individuals are essential as a source of infection. However, it is difficult to identify asymptomatic-infected individuals in the population, which makes an isolation approach less effective for controlling dengue fever. Promoting epidemic and sero-epidemiological registration at a larger population scale would be useful, along with identifying asymptomatic-infected individuals using laboratory methods. In the past few years, the fastest spreading and most widespread dengue virus in Guangzhou was serotype 1 [21]. When a person is infected by different serotypes of the virus, the risk of developing DHF and DSS in severe cases will increase, although antibodies have a protective effect on human bodies [28,29]. This effect is defined as antibody-dependent enhancement (ADE). Based on the rate of symptomatic-infected and asymptomatic-infected cases, it is estimated that 1415 persons out of 10,000 individuals in Guangzhou could be infected by dengue virus. Approximately 96.71% of dengue infection is asymptomatic in Guangzhou, and an estimated 13.68% of the whole population could be asymptomatic-infected. Considering the high rate of asymptomatic dengue virus infection in the population, stricter surveillance is necessary, as one crucial dengue intervention measure, to reduce the chance of silent transmission and to prevent ADE when secondary infection occurs. If an individual person is detected as positive for dengue IgG or IgM antibodies, frequent surveillance should be undertaken, in the presence or absence of clinical manifestations. Simpler and more economic testing methods should be used to identify sero-positive individuals. Screening of residents in the epidemic season is also important to strengthen the management of asymptomatic infection detection and reporting.

One of the limitations of this study was that there were only eight cases identified as symptomatic. A low symptomatic infection may induce larger errors in the reported rate and the symptomatic infection rate. This is likely further evidence for the relatively low dengue prevalence in Guangzhou. Another limitation was that detailed dengue case reports recorded in Guangzhou were from 2000. If the patient forgot that he/she was infected dengue virus and was a symptomatic case before 2000, and the IgG remained positive when the blood was collected in this study between 2013 and 2015, the number of symptomatic case will be underestimated and the rate of asymptomatic infection may be higher than the true value. However, this situation is very rare because the dengue prevalence was low in Guangzhou before 2000. Additionally, recall bias and symptomatic cases who did not seek normal medical service and did not know from what disease they suffered, would also result in a classification as asymptomatic infection. To decrease recall bias, we compared and verified the subjects’ information with the national reporting system for notifiable infectious diseases. In fact, just those reported in the national reporting system for notifiable infectious diseases were treated as dengue cases. Therefore, from the view of public health, those who were probably symptomatic but were not reported and were considered as asymptomatic are the same as those who were really asymptomatic.

On the other hand, the main strength of this study is that this is a relatively large-scale community-based study compared with other similar studies based on close contacts of the dengue fever patients, providing a relative comprehensive understanding of dengue prevalence in the general community population.

## Figures and Tables

**Table 1:** Demographic distribution of IgG and IgM

Variables	Number	Dengue IgG				Dengue IgM			
	(Percentage %)	Positive Number	Positive rate % (95% CI)	*p*-value	Odds ratio (95% CI)	Positive Number	Positive rate % (95% CI)	*p*-value	Odds ratio (95% CI)
Total number	2085	246	11.80 (10.41–13.18)			83	3.98 (3.14–4.82)		
Gender				0.212				0.575	
Male	764 (36.6)	99	12.96 (10.57–15.34)		1 (referent)	28	3.66 (2.33–5.00)		1 (referent)
Female	1321 (63.4)	147	11.13 (9.43–12.83)		0.84 (0.64–1.10)	55	4.16 (3.08–5.24)		1.14 (0.72–1.82)
Age (years old)				0.000				0.399	
<19	416 (20)	7	1.68 (0.44–2.92)		1 (referent)	18	4.33 (2.36–6.30)		1 (referent)
19–40	433 (20.8)	22	5.08 (3.00–7.16)		3.13 (1.32–7.40)	14	3.23 (1.56–4.91)		0.73 (0.36 –1.51)
41–65	879 (32.6)	48	7.07 (5.14 –9.00)		4.45 (1.99 –9.92)	33	4.86 (3.24 –6.48)		
>65	557 (26.7)	169	30.34 (26.51 –34.17)		25.45 (11.80 –54.89)	18	3.23 (1.76 –4.70)		0.74 (0.38 –1.44)
Educational status				0.000				0.081	
Illiterate	159 (7.6)	23	14.47 (8.94–19.99)		1 (referent)	6	3.77 (0.78–6.77)		1 (referent)
Primary	437 (21)	71	16.25 (12.78–19.72)		1.15 (0.69–1.91)	18	4.12 (2.25–5.99)		1.1 (0.43–2.81)
Junior high school	426 (20.4)	59	13.85 (10.56–17.14)		0.95 (0.56–1.60)	15	3.52 (1.76–5.28)		0.93 (0.35–2.44)
Senior high school	497 (23.8)	41	8.25 (5.82–10.68)		0.53 (0.31–0.92)	30	6.04 (3.94–8.14)		1.64 (0.67–4.01)
Diploma and above	247 (11.8)	20	8.1 (4.67–11.52)		0.52 (0.28–0.98)	8	3.24 (1.02–5.46)		0.85 (0.29–2.51)
Unknown	319 (15.3)	32	10.03 (6.72–13.35)		0.66 (0.37–1.17)	6	1.88 (0.38–3.38)		0.49 (0.16–1.54)
Marital Status				0.000				0.340	
Single	534 (25.6)	14	2.62 (1.26–3.98)		1 (referent)	19	3.56 (1.98–5.13)		1 (referent)
Married	1270 (60.9)	177	13.94 (12.03–15.84)		6.01 (3.46–10.47)	58	4.42 (3.28–5.55)		1.3 (0.76–2.20)
Widowed	121 (5.8)	36	29.75 (21.49–38.02)		15.73 (8.14–30.39)	2	1.65 (0.45–5.83)		0.46 (0.10–1.98)
Divorced	18 (0.9)	3	16.67 (5.84–39.22)		7.43 (1.93–28.61)	1	5.56 (0.99–25.76)		1.59 (0.20–12.61)
Unknown	142 (6.8)	16	11.27 (6.00–16.53)		4.72 (2.24–9.92)	3	2.11 (0.72–6.03)		0.59 (0.17–2.00)

Significance difference where *p*-value is less than 0.05. 95% confidence interval

**Table 2:** Detection results for different time periods

Antibody	Time Period	Guangzhou				
		Number	Positive Number	Positive rate % (95% CI)	*p*-Value	Odds ratio (95% CI)
IgG	09/2013–12/2013	349	37	10.60 (7.66–14.12)	0.011	1 (referent)
	01/2014–08/2014	871	95	10.91 (8.95/13.09)		1.03 (0.69–1.54)
	09/2014–12/2014	353	60	17.00 (13.32–21.15)		1.73 (1.11–2.68)
	01/2015–08/2015	512	54	10.55 (8.08–13.40)		0.99 (0.64–1.55)
IgM	09/2013–12/2013	349	7	2.01 (0.87–3.84)	0.000	1 (referent)
	01/2014–08/2014	871	20	2.30 (1.44–3.43)		1.15 (0.48–2.74
	09/2014–12/2014	353	33	9.35 (6.61–12.68)		5.04 (2.20–11.55)
	01/2015–08/2015	512	23	4.49 (2.92–6.52)		2.30 (0.98–5.42)

95% confidence interval. Significance difference between four time periods where the *p*-value of the antibody positive rate in Guangzhou is less than 0.05.
